# Sleep and personality in college students: A preliminary study

**DOI:** 10.1192/j.eurpsy.2021.1476

**Published:** 2021-08-13

**Authors:** A.P. Amaral, C. Fernandes, M. Pocinho

**Affiliations:** Coimbra Health School, Polytechnic of Coimbra, Coimbra, Portugal

**Keywords:** Personality, College students, sleep quality, Emotionality

## Abstract

**Introduction:**

Sleep represents an important process in the stable behavioural and emotional functioning of the individual and is an important health indicator. Personality is related with academic and occupational achievement, quality of interpersonal relationships, but also with sleep. Concerning personality, individuals with lower emotional stability have greater sensitivity to stress and maladaptive sleep-related behaviour.

**Objectives:**

The main goal of this study is to analyze the relation between sleep quality and personality in college students.

**Methods:**

This study employed a correlational design. A sample of 220 Portuguese students (84.9% females), with mean age of 19.5 years (sd=3.4), from different health courses, filled in the Pittsburgh Sleep Quality Questionnaire and HEXACO-60, during a single individual session. A descriptive statistical analysis, a Pearson correlation analyses and the t Student test, for independent samples, were performed.

**Results:**

The results showed a predominance of poor sleep quality among students (96.3%). The more prevalent HEXACO dimensions are: Conscientiousness (X=32.6; sd=4.2) and Emotionality (X=31.2; sd=5.2). When exploring personality differences between sleep groups (GSG=Good Sleep Group; PSG=Poor Sleep Group) a significant difference was found in mean scores of the dimension Emotionality. It was observed that the PSG revealed higher levels of Emotionality than the GSG (PSG=31.5; sd=5.1; PSG=26.3; sd=4.0; p<.05).

**Conclusions:**

College students self-report a poor sleep and the prevalent personality dimensions are Conscientiousness and Emotionality. Students with higher levels of Emotionality (fearfulness, anxiety, dependence and sentimentality) presented a poor sleep. To conclude, mediation studies are needed in order to better understanding the link between personality and sleep.

